# Trace glucose and lipid metabolism in high androgen and high-fat diet induced polycystic ovary syndrome rats

**DOI:** 10.1186/1477-7827-10-5

**Published:** 2012-01-25

**Authors:** Hua-Ling Zhai, Hui Wu, Hui Xu, Pan Weng, Fang-Zhen Xia, Yi Chen, Ying-Li Lu

**Affiliations:** 1 Endocrinology and Metabolism Research Institute and Department of Endocrinology and Metabolism, Shanghai Ninth People's Hospital Affiliated Shanghai Jiaotong University School of Medicine, Shanghai 200011, China

**Keywords:** Andronate, Glucose metabolism, Lipid metabolism, High-fat diet, Polycystic ovary syndrome

## Abstract

**Background:**

There is a high prevalence of diabetes mellitus (DM) and dyslipidemia in women with polycystic ovary syndrome (PCOS). The purpose of this study was to investigate the role of different metabolic pathways in the development of diabetes mellitus in high-androgen female mice fed with a high-fat diet.

**Methods:**

Female Sprague-Dawley rats were divided into 3 groups: the control group(C), n = 10; the andronate-treated group (Andronate), n = 10 (treated with andronate, 1 mg/100 g body weight/day for 8 weeks); and the andronate-treated and high-fat diet group (Andronate+HFD), n = 10. The rate of glucose appearance (Ra of glucose), gluconeogenesis (GNG), and the rate of glycerol appearance (Ra of glycerol) were assessed with a stable isotope tracer. The serum sex hormone levels, insulin levels, glucose concentration, and the lipid profile were also measured.

**Results:**

Compared with control group, both andronate-treated groups exhibited obesity with higher insulin concentrations (*P *< 0.05) but similar blood glucose concentrations. Of the two andronate-treated groups, the andronate+HFD group had the most serious insulin resistance (IR). Estrus cycles were completely acyclic, with polycystic ovaries and elevated serum lipid profiles in the andronate+HFD group (*P *< 0.05). Ra of glucose and GNG increased significantly in the andronate+HFD rats. However, the Ra of glycerol was similar in the three groups.

**Conclusions:**

Andronate with HFD rat model showed ovarian and metabolic features of PCOS, significant increase in glucose Ra, GNG, and lipid profiles, as well as normal blood glucose levels. Therefore, aberrant IR, increased glucose Ra, GNG, and lipid metabolism may represent the early-stage of glucose and lipid kinetics disorder, thereby might be used as potential early-stage treatment targets for PCOS.

## Background

Polycystic ovary syndrome (PCOS) is one of the most common endocrine disorders in women of reproductive age [1] and is the most frequent cause of hyperandrogenism and anovulation [2]. PCOS is also strongly associated with abdominal obesity, hyperinsulinemia, insulin resistance, and type 2 diabetes [3]. The pathophysiology of PCOS is largely unknown but has been attributed to defects in various organ systems. Uncontrolled ovarian steroidogenesis with a thickened thecal layer that secrets excessive androgen is thought to be a primary abnormality of PCOS [4]. PCOS is combined with defects in insulin action and insulin resistance (IR) finally leading to diabetes, and it also displays neuroendocrine dysfunction with exaggerated LH pulsatility, and altered production of adrenal androgen [5].

Once a diagnosis of PCOS is confirmed, it is imperative to assess the woman for diabetes mellitus (DM) risk factors. Despite the many reasons that women seek medical care for PCOS, the greatest long-term risk for these women is DM [6]. The link between PCOS and DM is multi-faceted [7]. Insulin resistance (IR) is increased in age-matched PCOS women and is linked to hyperandrogenism [8]. No single blood test is available to predict or measure this DM risk. Although no consensus recommendation for the assessment of DM risk factors exists, measurement of glucose metabolism, lipid screening, and measurements of insulin concentrations have been suggested [9].

Due to the heterogeneity of PCOS, it is difficult to create a single animal model that expresses the main PCOS characteristics [10]. We used andronate (testosterone propionate), a steroid hormone of the androgen group, in combination with high-fat diet (HFD) to establish a rat model of PCOS. The aim of this study was to investigate the metabolic pathways in glucose metabolism, lipid production, and gluconeogenesis with a stable tracer method. This method allows noninvasive detection of key steps in glucose and lipid metabolism, leading to the understanding of the mechanisms by which PCOS modifies glucose and lipid metabolism and treatment targets for PCOS.

## Methods

### Animals and experiment design

**Figure 1:**
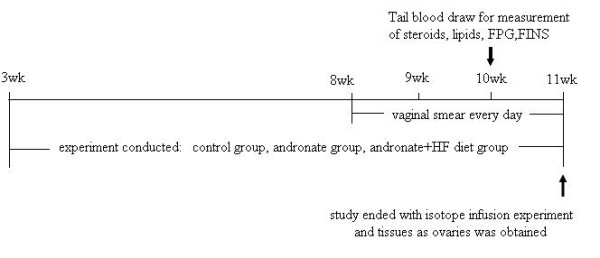
**Research protocol of the experiment**. The rats were divided into three groups. The arrows indicate the times of blood draws and isotope infusion. A vaginal smear was administrated everyday since 8 weeks.

Female Sprague-Dawley rats (3 weeks old) were bred and housed locally at 22°C ± 2°C under a 12 h on, 12 h off light cycle with free access to food and water. The rats were randomly divided into three groups for the next 8 weeks of treatment. The protocol of the study is presented in Figure 1.

Control group (n = 10): The rats were fed with a standard laboratory diet (52% carbohydrate, 22.1% protein, 9.2% water, 5.28% fat, 4.12% cellulose, 4.22% mineral salts) and were injected with olive oil of a similar volume as the experimental group.

Andronate group (n = 10): The rats were injected with andronate subcutaneously at a dose of 1 mg/100 g body weight/day for 8 weeks. The rats were also fed with a standard laboratory diet.

Andronate combined high-fat diet group (n = 10): The rats were injected with andronate subcutaneously at a dose of 1 mg/100 g body weight/day for 8 weeks. The rats were also fed with a lipid-enriched diet (54.2% standard diet, 16.8% lard, 15% sucrose, 9% casein, 1% minerals, 1% vitamins, 3% malt dextrin).

Body weight and blood glucose were measured weekly. All the animal procedures were performed in accordance with the ethical principles in animal research adopted by the Department of Laboratory Animal Science, Jiaotong University School of Medicine, Shanghai, China.

### Vaginal smears

A moistened cotton bud swab was gently inserted into the vagina. The cells were removed from the vaginal lumen and walls and then transferred onto a glass slide. It is the quickest way of smearing and the smears retain their original appearance indefinitely. The stage of cyclicity was determined by microscopic analysis of the predominant cell types in vaginal smears. The vaginal smears were analyzed daily since 8 weeks of age to the end of the experiment.

### Ovarian morphology

Ovarian tissue from each group was fixed in 4% paraformaldehyde, dehydrated with ethanol and xylene, embedded in paraffin, and sliced into 5-μm sections on a microtome (SLEE, Germany). The sections were stained with hematoxylin-eosin (HE) and analyzed under an optical microscope (CKX41, Olympus, Japan).

### Blood sample collection and assays

One week prior to the treatment, tail blood was obtained after an overnight fast to assess the glucose, insulin, and sex steroid levels and the lipid profile. Plasma glucose and lipids concentration were assayed by Siemens Dimension MAX (Siemens Healthcare Diagnostics Inc). Plasma insulin was assayed by magnetic affinity immunoassay (Insulin MPAIA Kit). Sex steroids, including follicle-stimulating hormone (FSH), luteinizing hormone (LH), and testosterone (T) were assayed by chemiluminescent microparticle immunoassay (CMIA).

### Stable isotope infusion procedure

**Figure 2:**
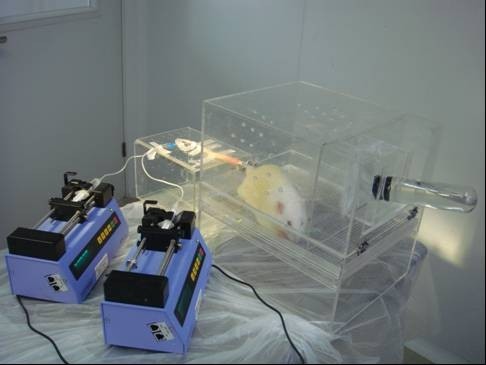
**Experimental setting for stable isotope infusion**. The rats catheterized in the tail artery for blood collection and in the tail vein for isotope infusion. The rats were relaxed, with the ability to move partially, groom and drink. Thus, experimental stress was minimized.

At 11 weeks of age, the tail-artery was catheterized for blood collection after an overnight fast. During the procedure, only the tail was anesthetized locally with lidocaine. The procedure took 15 min to complete. This catheterization allowed frequent collection of arterial blood samples while tracers were infused intravenously, as well as the adherence to the optimal V-A mode of metabolic experiments. The animals were relaxed with the ability to move partially, groom and drink. Thus, experimental stress was minimized. When blood glucose returned to baseline (usually within 30 min) shown by a hand-held glucose device (Terumo, medisafe mini blood glucose reader, manufactured by: Terumo corporation, Tokyo, Japan), a flexible plastic intravenous infusion line was placed in the lateral tail vein by venipuncture, and isotopic tracer infusions were commenced as described below. The experimental setting is shown in Figure 2.

### In vivo experiments

**Figure 3:**
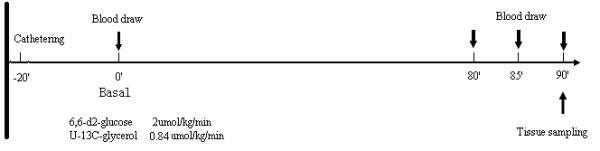
**Protocol of in vivo infusions of stable isotopes**. This is the protocol of steady-state isotope infusion. The arrows indicate the times for blood draws and tissue sampling.

In this study, appearance of glucose and glycerol were traced by [6,6-2D]-glucose and [U-13 C]-glycerol, while gluconeogenesis (GNG) were accessed by [1,2,3-13 C]-glucose and [U-13 C]-glycerol, [6,6-2D]-glucose (2 μmol/kg/min) and [U-13 C]-glycerol (0.84 μmol/kg/min) were infused intravenously constantly, without priming, through the tail infusion line driven by an infusion pump (Harvard Apparatus, Holliston, MA, USA)for 90 min. This is defined as the basal period. During the last 10 min (80-90 min), arterial blood samples (0.5 ml each) were collected 5 min intervals from the tail arterial catheter. These samples were used for the quantitation of steady state glucose and glycerol metabolism. The rats were then euthanized by heart-opening under anesthesia with pentobarbital (50 mg/kg) in order to reduce blood elements in tissues. Ovaries were harvested swiftly. Plasma was prepared on site and saved at -80°C for later analysis. A flow chart of the study design is shown in Figure 3.

### Gas chromatography/mass spectrometry

Plasma samples from the infusion experiments were processed chromatographically by methoxyamine-HCl and BSTFA to get m/z: 321/319 [6,6-2D]-glucose, and m/z: 221/218 [U-13 C]-glycerol. To exclude the interference of spill over from the [6,6-2D]-glucose to [1,2,3-13 C]-glucose (formed in gluconeogenesis whose precursor is [U-13 C]-glycerol), hydroxylamine hydrochloride was used to derivation. Then the derivation was analyzed by gas chromatography/mass spectrometry (autoSystem XL GC/TurboMass MS) for the enrichment. The GC oven temperature was programmed initially at 70°C for 4 min, increased to 240°C at 10°C/min, then to 300°C at 20°C/min, and stayed at 300°C for 11 min. The GC was electronically controlled for constant pressure and humidity.

### Calculation of glycerol and glucose appearance rates and rates of gluconeogenesis

The infusion rate (μmol/kg/min) of [U-13 C]-glycerol and [6,6-2D]-glucose was divided by mole percent excess (MPE) of plasma glycerol and that of glucose, respectively, to give the appearance rates (μmol/kg/min).

Gluconeogenesis rates were calculated by dividing the MPE of plasma [U-13 C]-glycerol by the MPE of [1,2,3-13 C]-glucose. In this experiment, appreciable amounts of [U-13 C]-glycerol were converted to [1,2,3-13 C]-glucose via gluconeogenesis. Thus gluconeogenesis rates can be compared among different groups.

### Statistical analysis

The data were expressed as mean ± standard deviation (SD), unless otherwise indicated. Statistical significance was tested by analysis of variance (ANOVA). A value of *P <*0.05 was considered significant.

## Results

### Body weight and blood glucose curves

**Figure 4:**
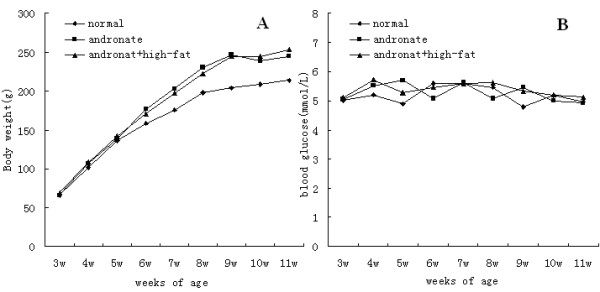
**Body weight and blood glucose levels in three groups**. Since 3 weeks of age, body weight measurements and blood glucose levels were taken every week. **A**: body weight changes in the 3 groups; **B**: blood glucose levels in the 3 groups.

Andronate-treated two groups exhibited a significant increase in the mean body weight at the end of the study (andronate group: 244.5 ± 11.5, andronate + HFD group: 253.8 ± 19.7; vs. control group: 214.7 ± 13.0, *P *< 0.05) (Figure 4A). However, there was no significant difference in blood glucose levels between the three groups over the 8 weeks time course (Figure 4B).

### Reproductive cycles of rats

**Figure 5:**
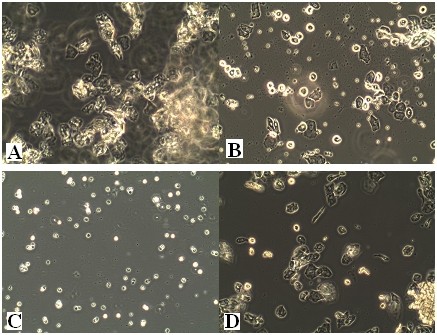
**The main stages of the reproductive cycle in the control rats**. Swab smears (unstained) shown with the original microscope magnification of 100×. **A**:Estrus stage: large cornified cells in clumps. **B**: Metestrus stage: large numbers of leucocytes with smaller numbers of non-nucleated epithelial cells (note characteristic clumping together of two cell types at the center-right). **C**: Diestrus stage: predominantly leucocytes with a small number of epithelial and cornified cells. **D**: Proestrus stage:epithelial cells are mostly rounded but some cells shows early stages of cornification of approaching estrus.

Reproductive cycles were assessed by vaginal smears. The control group exhibited regular estrous cycles (Figure 5), whereas the two experimental groups had prolonged or irregular estrous cycles, most of which showed a constant diestrus smear (Figure 5C).

### Ovarian morphology

**Figure 6:**
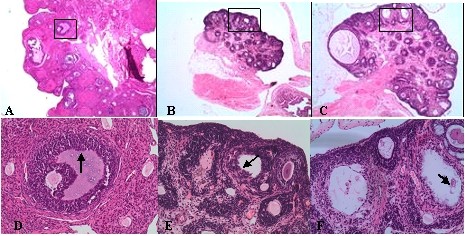
**Histology of ovaries**. Ovaries were stained by HE staining:**A**:control group(HE staining, ×40); **B**:andronate group(×40); **C**:andronate + HFD group(×40); **D**:control group(×200); **E**:andronate group(×200); **F**:andronate + HFD group(×200).

Light microscope revealed normal ovarian structures in the control group (Figure 6A, D), with follicles and corpora lutea in various stages and no ovary cysts were observed. The ovaries of the andronate-treated group were much smaller than those of the control group (Figure 6B), with many cystic follicles with apoptotic granulosa cells (Figure 6E). The andronate + HFD group had larger ovaries than the control group, with a fatty infiltration surrounded the ovaries (Figure 6C). In addition, cystic follicles with macrophages and fluid were also observed (Figure 6F). In general, both experimental groups had fewer corpus lutea and preovulatory follicles and had more preantral and antral follicles than the controls.

### Body hair changes

**Figure 7:**
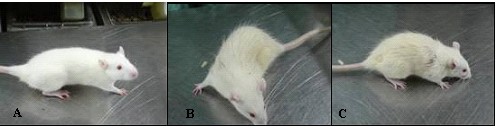
**Body hair in the three rats groups**. At 10 weeks of age, pictures were taken of the 3 groups. **A**:control group; **B**:andronate group; **C**:andronate with HFD group.

After 8 weeks treatment with andronate and diet changing, the two treated groups showed hirsutism and longer body hair, while the control group had no unusual body hair changes (Figure 7).

### Fasting blood glucose (FBG) levels, fasting plasma insulin (FINS) levels, homeostasis model-insulin resistance (HOMA-IR), and insulin sensitivity index (ISI)

**Figure 8:**
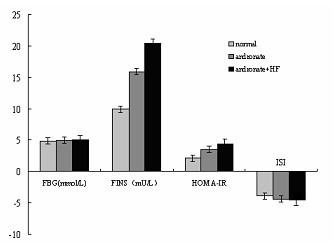
**FBG, FINS, HOMA-IR and ISI in the 3 groups**. Tail blood was obtained after an overnight fast in 10-week-old rats to assess fasting blood glucose (FBG), fasting plasma insulin (FINS), homeostasis model-insulin resistance (HOMA-IR) and insulin sensitivity index(ISI) in 3 groups.

The FBG levels were similar among all the three groups, approximately 5 mmol/L. The FINS concentrations were 10.0 ± 1.1 mU/L (control), 15.9 ± 2.1 mU/L (andronate), and 20.3 ± 1.8 mU/L (andronate and HF), respectively. The HOMA-IR in the andronate + HFD group was markedly elevated (4.43 ± 0.45), while was mildly elevated in the andronate group (3.49 ± 0.49) compared to the control group (2.14 ± 0.21). The insulin sensitivity index was inverse, as both the andronate + HFD and the andronate groups had a lower insulin sensitivity index (-4.6 ± 0.10 and -4.35 ± 0.15, respectively) than the control group (-3.87 ± 0.10, *P <*0.05) (Figure 8).

### Profiles of serum hormone levels

Although FSH levels were not affected, the LH levels were increased in the andronate-treated groups. Consequently, the LH/FSH ratio was elevated in the treated group, mirroring the changes observed in human PCOS. Furthermore, testosterone levels were significantly augmented in the andronate + HFD group, indicating that hyperandrogenism was present in this animal model (Table 1).

**Table 1:** FSH, LH, LH/FSH and T of andronate treated and control groups

Group	Control	Andronate	Andronate+high-fat diet
FSH(mIU/ml)	0.018 ± 0.0075	0.017 ± 0.0082	0.017 ± 0.0052
LH(mIU/ml)	0.045 ± 0.0014	0.058 ± 0.0021^a^	0.053 ± 0.016^a^
LH/FSH	2.61 ± 0.61	3.75 ± 1.17^a^	3.25 ± 0.52^a^
T(ng/ml)	2.37 ± 0.23	14.33 ± 0.92^b^	16.35 ± 1.55^b^

Values expressed as mean ± SD, a: *p *< 0.05, b: *p *< 0.01

### Plasma lipid profile

Disturbances in the plasma lipid profiles were apparent (Table 2). There were increases in plasma concentrations of TC (total cholesterol), TG (triglycerides), and LDL-C (high density lipoprotein-cholesterol) in andronate-treated rats, and the plasma concentrations of these molecules were further elevated in the andronate + HFD group. Plasma HDL-C (high density lipoprotein -cholesterol) levels also tended to be elevated in the two groups, but the difference was not significant.

**Table 2:** TG, TC, HDL-C and LDL-C of andronate treated and control groups

Group	Control	Andronate	Andronate +high-fat
TG(mmol/L)	0.80 ± 0.11	1.06 ± 0.13^a^	1.24 ± 0.17^b^
TC(mmol/L)	1.51 ± 0.15	1.88 ± 0.15^a^	2.32 ± 0.16^b^
HDL-C(mmol/L)	0.56 ± 0.10	0.57 ± 0.09	0.61 ± 0.10
LDL-C(mmol/L)	0.46 ± 0.10	0.50 ± 0.09	0.62 ± 0.05^a^

Values expressed as mean ± SD, a: *p *< 0.05, b: *p *< 0.01

### Rates of glucose appearance (glucose Ra), gluconeogenesis (GNG), rates of glycerol appearance (glycerol Ra)

The glucose Ra was significantly increased in the andronate-treated groups, while that of glycerol was not affected. Fractional gluconeogenesis was most elevated in the andronate + HFD group, indicating that the combination of andronate and high-fat diet can increase body glucose by accelerating gluconeogenesis from glycerol (Table 3).

**Table 3:** Glucose, glycerol Ra, GNG, MPE of U-C13-glycerol and 1,2,3-C13-glucose in andronate treated and control groups

Group	Control	Andronate	Andronate +HF
Glucose Ra (umol/kg/min)	52.7 ± 9.6	73.8 ± 12.4^a^	79.4 ± 11.5^a^
Glycerol Ra(umol/kg/min)	17.3 ± 6.2	17.8 ± 3.4	16.5 ± 4.5
GNG(%)	13.4 ± 1.5	16.7 ± 2.3^a^	18.5 ± 4.7^a^
Mpe of U-C13-glycerol	4.87 ± 0.68	5.03 ± 0.63^a^	5.34 ± 0.79^b^
Mpe of 1,2,3-C13-glucose	0.63 ± 0.12	0.82 ± 0.11^a^	0.97 ± 0.20^a^

Values expressed as mean ± SD, a: *p *< 0.05, b: *p *< 0.01

## Discussion

Metabolism Syndrome (MS) is estimated to affect approximately 40% of women with PCOS, leading to increased prevalence of hypertension, dyslipidemia, and abnormal glucose metabolism [11]. We here demonstrate that andronate with HFD rats replicate both the ovarian and the metabolic syndrome features of human PCOS, including PCO morphology, irregular cycles, increased body weight, hyperlipidemia, and insulin resistance.

It has been suggested that adolescent obesity is correlated with insulin resistance (IR), dyslipidemia, and PCOS related ovulatory dysfunction [12]. In the present study, obesity was observed in andronate-treated groups; however, there was no significant difference between the groups fed with normal or high-fat diets. These results were consistant to our previous study of castrated male rats [13], and is likely due to failed adaptation to HFD.

Up to 70% of women with PCOS are also insulin resistant (IR), and the prevalence of DM in women with PCOS is 10% [14]. IR is a state of impaired metabolic response to insulin as characterized by the American Diabetes Association (ADA) [15]. The probable mechanisms of insulin-related reproductive abnormalities include excessive LH secretion, abnormalities of ovarian steroidogenesis, and abnormal glucose uptake in PCOS [16]. Although some women with PCOS initially exhibit normal glucose metabolism, the conversion rate of abnormal glucose metabolism in 3 years is 25% [17].

This study describes an apparent insulin resistance with increased HOMA-IR and decreased ISI in the andronate-treated group fed with HFD, though blood glucose (BG) of the three groups remains within normal range. Therefore, although the glucose metabolism abnormalities did not affect the blood glucose levels, the potential insulin resistance might have already existed. Thus, blood glucose level alone can not predict metabolic risk in PCOS, and the precursor states of insulin abnormalities may predict the risk of DM well before BG abnormalities arise [18].

The abnormalities of glucose and lipid metabolism were evaluated with stable isotopes. The results suggested a higher rate of glucose appearance (Ra of glucose) and gluconeogenesis (GNG) in andronate + HFD rats; though the rate of glycerol appearance (Ra of glycerol) showed no differences between the three groups. Ra of glycerol provides a good measurement of whole-body lipolysis during fasting. Because triacylglycerol is broken down into glycerol and three fatty acids, the amount of fatty acids released into circulation is three times the rate of glycerol production. Therefore, although this study showed no significant difference in the Ra of glycerol among the three groups, the lipid profiles change significantly in the andronate-treated goups. The increased Ra of glucose and GNG in combination with the unaffected BG indicate that the dynamics of glucose metabolism have been actively initiated in the early state, which maybe compensated increased to antagonist the elevated plasma insulin.

For the past several years, a number of PCOS rat models have been used to study the etiology and pathophysiology of PCOS. However, all of these methods have their limitations. As PCOS can be induced with estradiol valerate, it has been found that estradiol valerate results in acyclicity and PCOS-like ovarian morphology but does not cause the typical metabolic disturbances of human PCOS [19]. In another promising rat model, PCOS is induced by letrozole, an aromatase inhibitor that blocks the conversion of testosterone to estradial. While this model exhibits morphological similarities to human PCOS, it does not decrease insulin sensitivity despite increased testosterone concentrations [20]. After continuous exposure to dihydrotestosterone (DHT), rats develop PCOS characteristics, including increased number of apoptotic follicles, obesity, and insulin resistance. Thus, the DHT model is generally preferable for studies of both ovarian and metabolic features. However, in this model, the concentration of testosterone is low, and the mechanism of the endogenous production of androgens or testosterone is likely to be reduced by DHT treatment [21]. It has been strongly suggested that high-fat diet can induce liver insulin resistance [22] and that rats fed with HFD for 6 weeks are notably hyperinsulinemia. Therefore, andronate combined with high-fat diet to establish a rat model that manifests increased similarities in the ovary and metabolic features of PCOS.

The relationship between hyperandrogenism and IR is significant [23]. Many human research studies have demonstrated the high degree of this correlation [24]. Furthermore, the risk of metabolic syndrome in females with PCOS is highly correlated with increased testosterone concentrations [25]. A recent review demonstrated that women with elevated T concentrations have a higher risk of developing type 2 diabetes mellitus [26]. Animal model have also shown that prenatal testosterone exposure can induce IR in early postnatal life [27], which was confirmed in the present study.

## Conclusions

A rat model of PCOS was successfully established with features of polycystic ovaries, obesity, irregular cycles of vaginal smear, increased plasma insulin levels, decreased insulin sensitivity, hyperandrogenism, and increased LH concentrations. This animal model also exhibited significant increase in glucose Ra, rates of GNG, and lipid profiles despite having normal blood glucose levels. Therefore, we propose that IR, glucose Ra, GNG rates and lipid metabolism may be potential treatment targets for early-stage PCOS. Further studies are needed to evaluate the effects of these treatments on PCOS and to elucidate the complexity of PCOS etiology and pathophysiology.

## Abbreviations

DM: Diabetes mellitus; PCOS: Polycystic ovary syndrome; HFD: High-fat diet; Ra of glucose: Rate of glucose appearance; GNG: Gluconeogenesis; Ra of glycerol: Rate of glycerol appearance; IR: Insulin resistance; MPAIA: Magnetic particle antibody immunoassay; FSH: follicle-Stimutating hormone; LH: Luteinizing hormone; T: Testosterone; CMIA: Chemiluminescent microparticle immunoassay; GC: Gas chromatography; MPE: Mole percent excess; SD: Standard deviation; FBG: Fasting blood glucose; FINS: Fasting plasma insulin; HOMA-IR: Homeostasis model-insulin resistance; ISI: Insulin sensitivity index; TC: Total cholesterol; TG: Triglycerides; LDL-C: High density lipoprotein-cholesterol; HDL-C: High density lipoprotein-cholesterol; MS: Metabolism Syndrome; ADA: American diabetes association; DHT: Dihydrotestosterone.

## Competing interests

The authors declare that they have no competing interests.

## Authors' contributions

H-LZ has supervised the entire work on the animals, done the isotope analysis and wrote the manuscript. HW has helped the animal catheterized. HX has done the statistical analysis of data and PW has done the animal model construction including ovary morphology HE staining and vaginal smears. F-ZX has done the lipid assays and YC has done the insulin assays. Y-LL contributed to the concept of the study and has supervised all the work. All authors read and approved the final manuscript.

## Authors' information

Hua-Ling Zhai during the experiment was PhD student. She is now attending physician at Endocrinology and Metabolism Research Institute and Department of Endocrinology and Metabolism, Shanghai Ninth People's Hospital Affiliated Shanghai Jiaotong University School of Medicine. Hui Wu, Hui Xu, Yi Chen and Pan weng are all postgraduate students. Fang-Zhen Xia is a permanent research worker. Ying-Li Lu is professor and director of endocrinology department in Shanghai Ninth People's Hospital Affiliated Shanghai Jiaotong University School of Medicine.
